# Strategy to enhance efficacy of doxorubicin in solid tumor cells by methyl-β-cyclodextrin: Involvement of p53 and Fas receptor ligand complex

**DOI:** 10.1038/srep11853

**Published:** 2015-07-07

**Authors:** Naoshad Mohammad, Shivendra Vikram Singh, Parmanand Malvi, Balkrishna Chaube, Dipti Athavale, Muralidharan Vanuopadath, Sudarslal Sadasivan Nair, Bipin Nair, Manoj Kumar Bhat

**Affiliations:** 1 National Centre for Cell Science, Pune University Campus, Ganeshkhind, Pune- 411007, India; 2 Amrita School of Biotechnology, Amrita Vishwa Vidyapeetham University, Kollam-690525, India

## Abstract

Doxorubicin (DOX) is one of the preferred drugs for treating breast and liver cancers. However, its clinical application is limited due to severe side effects and the accompanying drug resistance. In this context, we investigated the effect on therapeutic efficacy of DOX by cholesterol depleting agent methyl-β-cyclodextrin (MCD), and explored the involvement of p53. MCD sensitizes MCF-7 and Hepa1–6 cells to DOX, Combination of MCD and marginal dose of DOX reduces the cell viability, and promoted apoptosis through induction of pro-apoptotic protein, Bax, activation of caspase-8 and caspase-7, down regulation of anti-apoptotic protein Bcl-2 and finally promoting PARP cleavage. Mechanistically, sensitization to DOX by MCD was due to the induction of FasR/FasL pathway through p53 activation. Furthermore, inhibition of p53 by pharmacological inhibitor pifithrin-α (PFT-α) or its specific siRNA attenuated p53 function and down-regulated FasR/FasL, thereby preventing cell death. Animal experiments were performed using C57BL/6J mouse isografted with Hepa1–6 cells. Tumor growth was retarded and survival increased in mice administered MCD together with DOX to as compared to either agent alone. Collectively, these results suggest that MCD enhances the sensitivity to DOX for which wild type p53 is an important determinant.

Breast and hepatocellular carcinoma (HCC) are the second and fifth most prevalent cancers respectively, and leading causes of cancer associated deaths in the entire world^1^,^2^,^3^. Although surgical removal of tumor is still the primary treatment of choice, apart from surgery or radiotherapy, chemotherapy remains to be most efficient way for preventing cancer cell growth and metastasis thereby enhancing the survival of cancer patients^4^. One of the major limitations of chemotherapeutic drugs is toxicity due to high dose regimen or improper efficacy of drugs towards tumor cells^5^. Therefore, new strategies to achieve favorable response to chemotherapy for improvement in the prognosis of breast and liver cancer are urgently desirable.

Doxorubicin (DOX), an anthracycline antibiotic, is one of the most effective and widely used chemotherapeutic agents for the treatment of various malignancies including breast and liver for the past twenty years^6^. However, the common drawbacks in the clinical use of DOX are cardiotoxicity and bone marrow depression at higher doses^7^. DOX induces apoptosis in cancer cells by DNA damage, generation of reactive oxygen species, cell cycle arrest and activation of p53^8^,^9^,^10^,^11^,^12^. Various studies have shown that the expression of wild-type p53 is essential for the cytotoxic response to chemotherapeutic agents. As the guardian of genome, the tumor suppressor p53 is activated upon DOX treatment and functions as a transcription factor thereby regulating downstream target genes such as BAX, PUMA and MDM2^13^,^14^,^15^. In this context, a couple of novel combination regimens have been found to be better suited for the treatment of cancers without inducing side effects to normal tissues^16^,^17^. Attempts have been made to identify chemosensitizing agents which could enhance the efficacy of DOX, and thereby reducing the DOX doses. Various agents such as curcumin, IFN-α, quercetin, selenocystine and ocotillol were studied to potentiate the antitumor activity of DOX via p53 activation^18^,^19^,^20^,^21^,^22^.

The drug delivery techniques specifically for cancer cells have received considerable attention in recent years. In this study, we have utilized cyclodextrin (CD) which are produced by starch through enzymatic reaction. Among all types of cyclodextrin, methyl β-cyclodextrin (MCD) a cyclic heptasaccharide consisting of exterior hydrophilic and interior hydrophobic cavities^23^,^24^. MCD is most accessible and extensively used in pharmaceutical industries as well as in biological researches because it augments the solubility, delivery and bioavailability of many molecules including drugs. It is the most effective agent for removal of plasma membrane cholesterol due to its high affinity towards it^25^. We have previously reported that MCD enhances the therapeutic efficacy of 5-flurouracil, carboplatin and tamoxifen^26^,^27^. Additionally, other studies also reported that MCD or its modified forms can increase the cytotoxic effect of various drugs^28^,^29^. In this study, we examined the ability of MCD to enhance the therapeutic efficacy of DOX in breast and liver cancer cells both by *in vitro* as well as *in vivo* studies. Our results demonstrate that combination of MCD and DOX reduces cell proliferation by promoting apoptosis. Mechanistically MCD acts as a potential chemosensitizer by enhancing DOX induced cell death through activation of p53 and induction of FasR/FasL pathway.

## Results

### Methyl β-cyclodextrin potentiates doxorubicin-induced cytotoxicity in MCF-7 and Hepa1–6 cells

**Figure 1:**
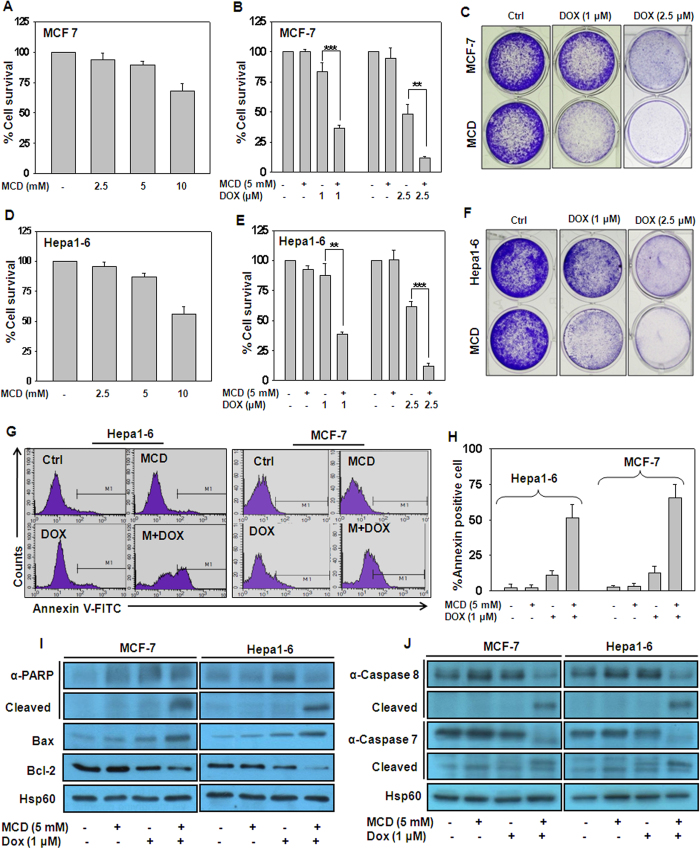
MCD potentiates the effect of DOX and induces apoptosis in breast and liver cancer cells. (**A**) MCF-7 and (**D**) Hepa1–6 cells were treated with indicated concentrations of MCD (2.5–10 mM) for 4 h and the cell viability was measured by MTT assay. (**B**) MCF-7 and (**E**) Hepa1–6 cells were treated with indicated concentrations of DOX together with MCD for 24 h and subjected to MTT assay. (**C**) MCF-7 and (**F**) Hepa1–6 cells were treated with indicated concentrations of DOX together with MCD for 24 h and cells were subjected to long term clonogenic assay. (**G**) MCF-7 and Hepa1–6 cells were treated with indicated concentration of DOX together with MCD for 24 h, and apoptotic cells were analyzed by Annexin V-FITC staining using flow cytometry. (**H**) Quantitation of Annexin V-FITC positive cells. (**I**) MCF-7 and Hepa1–6 cells were treated with indicated concentration of DOX together with MCD for 24 h. Cells were harvested, whole cell lysates were subjected to immunoblotting and the protein levels of PARP, Bax and Bcl-2 was assessed. Hsp60 served as a loading control. (**J**) MCF-7 and Hepa1–6 cells were treated with indicated concentration of DOX together with MCD for 24 h. Cells were harvested, whole cell lysates were subjected to immunoblotting and the protein levels of caspase-8 and caspase-7 and there cleaved form were assessed. Hsp60 served as a loading control. Cropped blots are used in the main figure and full length blots are included in Supplementary figure 1. All the bar graph represents the mean ± SD of an experiment done in triplicate (*P ≤ 0.05, **P ≤ 0.001, ***P ≤ 0.0001).

To investigate whether MCD has any adverse effect on MCF-7 and Hepa1–6 cells, screening experiments were performed to determine the non-toxic concentration and optimum time point of MCD suitable for use in combination treatment. Treatment of cells with various concentration of MCD (2.5 mM to 10 mM) for 4 h inhibited the cell survival in a dose-dependent manner as measured by MTT assay (Fig. 1A,D). MCD at 10 mM dose was highly toxic to cells as compared to 2.5 and 5 mM, hence, 5 mM concentration was used for further experiments. Additionally, the marginal dose of DOX for use in the combination regimen in cells treated with DOX was calculated to be 2.5 μM for both the cells (data not shown). Since DOX is used for the treatment of breast and HCC, it is necessary to define an approach to enhance the therapeutic index of DOX at lower doses. We investigated the combination effects of MCD on DOX induced effect in MCF-7 and Hepa1–6 cells. Cells were treated by MCD together with IC_50_ dose of DOX for 24 h. As anticipated, at IC_50_ dose of DOX, cell viability reduced by 50%, which was further reduced to less than 5% in the presence of MCD and these results were also verified by long term clonogenic assay (Fig. 1B,E,C,F). Next, we explored the effects of low dose (1 μM) of DOX together with MCD (5 mM) on viability of MCF-7 and Hepa1–6 cells and observed a significant reduction in cell survival as compared to MCD and DOX alone (Fig. 1B,E). Similar results were also observed in long term clonogenic assay (Fig. 1C,F). The increase in MCD potentiated DOX induced effect was due to induction of apoptosis in MCF-7 and Hepa1–6 cells as evident by graphical representation of FACS data and bar graph represents % Annexin positive cells (Fig. 1G,H). Consistent with above results combination treatment also caused PARP cleavage, decrease in the protein level of antiapoptotic protein Bcl-2, and up-regulation of proapoptotic protein Bax (Fig. 1I). Caspases play an important role in both intrinsic and extrinsic apoptotic pathways. Therefore, on examination of protein levels of caspases in MCF-7 and Hepa1–6, we found that MCD together with DOX activates caspase-8 and caspase-7 (Fig. 1J). All the above results demonstrate that MCD potentiates DOX induced cytotoxicity only in cancerous cell (MCF-7 and Hepa1–6) not in the normal hepatocytes (AML12) cells (Supplementary Figure 2A-D).

### MCD enhances intracellular accumulation of doxorubicin

**Figure 2:**
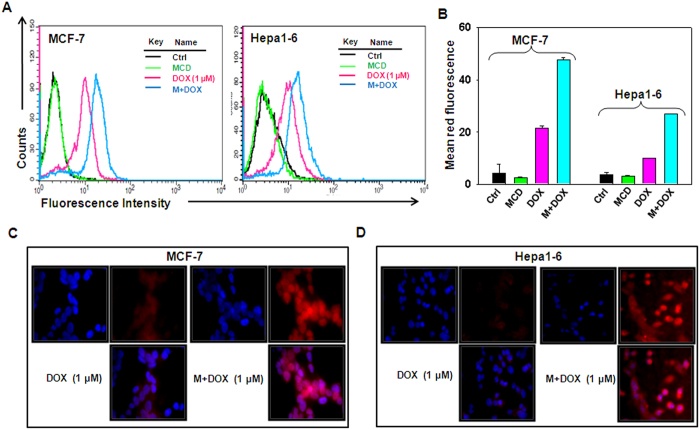
MCD augments intracellular uptake of DOX in MCF-7 and Hepa1–6 cells. MCF-7 and Hepa1–6 cells were treated with indicated concentration of DOX together with MCD. (**A**) Representative flow cytometric histogram of intracellular uptake of DOX in MCF-7 and Hepa1–6 cells. (**B**) Bar graph is representative of relative quantitation of DOX uptake in MCF-7 and Hepa1–6 cells. (**C**) MCF-7 and (**D**) Hepa1–6 cell were subjected to immunofluorescence confocal microscopy for the detection of intracellular uptake of DOX.

To explore the possible mechanism of MCD potentiated DOX induced cytotoxicity, we examined the effect of MCD on intracellular uptake of DOX using flow cytometry. The amount of DOX in the cell is directly proportional to its fluorescence because DOX is an auto-fluorescent drug. The measurement of fluorescence intensity of DOX has been used to evaluate cellular uptake in the cells^30^. Although DOX is taken up by cells as such, interestingly, following treatment with MCD, the cellular uptake of DOX was significantly enhanced as evident by graphical representation of FACS data and bar graph represents mean red fluorescence (Fig. 2A,B). Enhancement in the accumulation of DOX by MCD was further confirmed by fluorescence microscope (Fig. 2C,D). MCD did not potentiate DOX uptake in AML12 cells (Supplementary Figure 2E-F).

### MCD enhances the DOX induced p53 mediated upregulation of FasR/FasL in MCF-7 and Hepa1–6 cells

**Figure 3:**
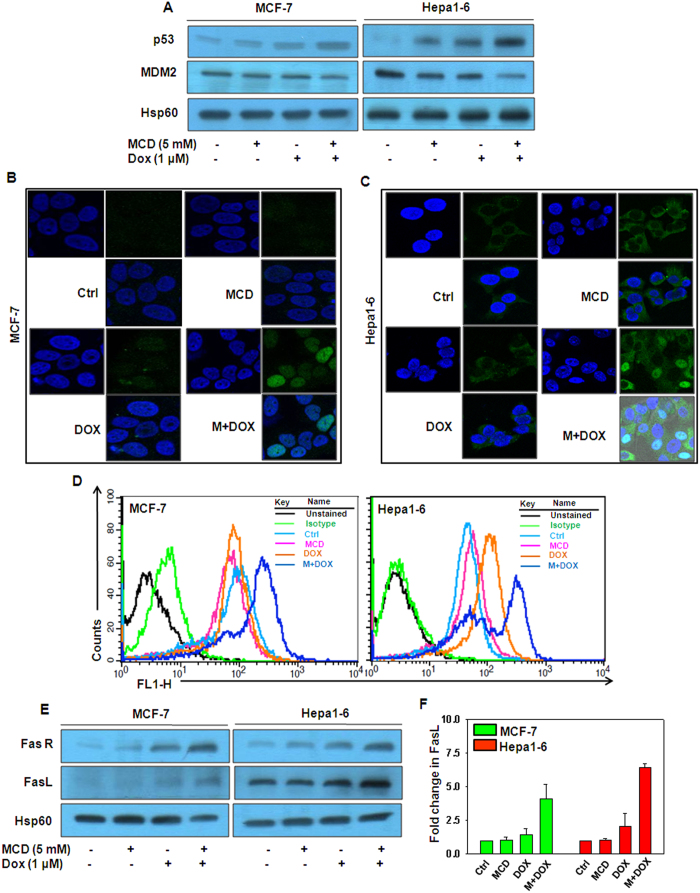
MCD potentiates DOX-induced death of MCF-7 and Hepa1–6 cells in a p53-dependent manner by upregulating FasR/FasL. MCF-7 and Hepa1–6 cells were treated with indicated concentration of DOX together with MCD for 24 h. (**A**) Protein levels of p53 and MDM2 were examined by western blotting analysis. Hsp60 served as internal control was used a loading control. (**B**) MCF-7 and (**C**) Hepa1–6 cells were processed for immunostaining to detect p53 expression and its nuclear localization by immunofluorescence confocal microscopy. (**D**) FasR membrane staining was performed by flow cytometry. (**E**) Protein levels of FasR and FasL were examined by western blotting analysis and Hsp60 was used a loading control. (**F**) Sandwich ELISA for quantitation of secreted FasL from culture medium of MCF-7 and Hepa1–6 cells. Bar graph represents the Mean ± SD of an experiment done in triplicate. Cropped blots are used in the main figure and full length blots are included in Supplementary figure 3.

Stress induced p53 promotes cell death and it is a key molecular mechanism of antitumor agents, such as DOX^31^. We explored the role of p53 in potentiating DOX induced cell death by MCD in MCF-7 and Hepa1–6 cells by western blot analysis. Relatively less p53 protein level was detected in cells treated with MCD and DOX alone, whereas combination treatment of MCD and DOX significantly increased the p53 protein level and decreased the protein level MDM2, a protein involved degrading p53 (Fig. 3A). Furthermore, increased p53 expression and its localization into nucleus of cells were confirmed by immunofluorescence confocal microscopy (Fig. 3B,C). Activation of p53 upon DNA damage leads to up-regulation of several proteins. Among these, death receptor protein, FasR has been shown to be upregulated in a number of cancer cells following genotoxic stress^32^,^33^,^34^. Additionally, p53 also augments the level of FasR on the plasma membrane by promoting trafficking of FasR from the Golgi^35^. The status of FasR was determined by flow cytometry and western blot analysis and we observed that DOX and MCD together significantly increased the expression of FasR as compared to either agent alone in MCF-7 and Hepa1–6 cells (Fig. 3D,E). Additionally, we also checked FasL protein level in cell lysates and its secreted form in the culture medium collected from the cells by western blot and by indirect ELISA respectively. The expression of FasL in whole cell lysates as well as in the culture medium was increased after treatment of DOX and MCD together (Fig. 3E,F).

### p53 abrogation reduces MCD potentiated DOX effect

**Figure 4:**
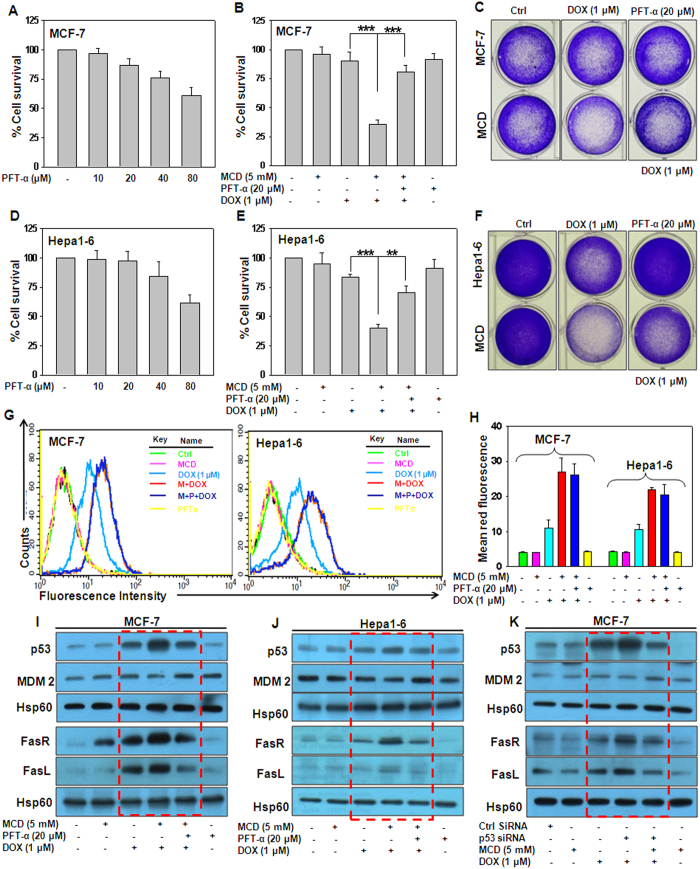
Silencing of p53 reduces MCD-sensitized DOX-induced cell death and down-regulation of FasR/ FasL. (**A**) MCF-7 and (**D**) Hepa1–6 cells were treated with varying concentrations of PFTα (10–80 μM) for 24 h and cell survival was evaluated by MTT assay. (**B**) MCF-7 and (**E**) Hepa1–6 cells were treated with indicated concentration of MCD, PFT-α and DOX for 24 h. Viability of cell were measured by MTT assay. (**C**) MCF-7 and (**F**) Hepa1–6 cells were treated with MCD, PFT-α and DOX as indicated and long term clonogenic assay was performed using crystal violet. (**G**) Representative flow cytometric histogram of intracellular uptake of DOX in MCF-7 and Hepa1–6 cells treated with MCD, PFT-α, and DOX. (**H**) Bar graph is representative of relative quantitation of DOX uptake in MCF-7 and Hepa1–6 cells. (**I**) MCF-7 and (**J**) Hepa1–6 cells were treated with indicated concentration of MCD, PFT-α and DOX for 24 h, the protein levels of p53, MDM2, FasR and FasL were examined by western blotting analysis. Hsp60 served as loading control. (**K**) MCF-7 cells were transfected with p53 siRNA as per manufacturer’s instructions. After 18 h of transfection, cells were exposed with MCD and DOX together for 24 h and whole cell lysates were prepared and immunoblotted for p53, MDM2, FasR and FasL. Hsp60 served as loading control. Bar graphs represent (Mean ± SD) experiments done in triplicate. Cropped blots are used in the main figure and full length blots are included in Supplementary figure 5.

**Figure 5:**
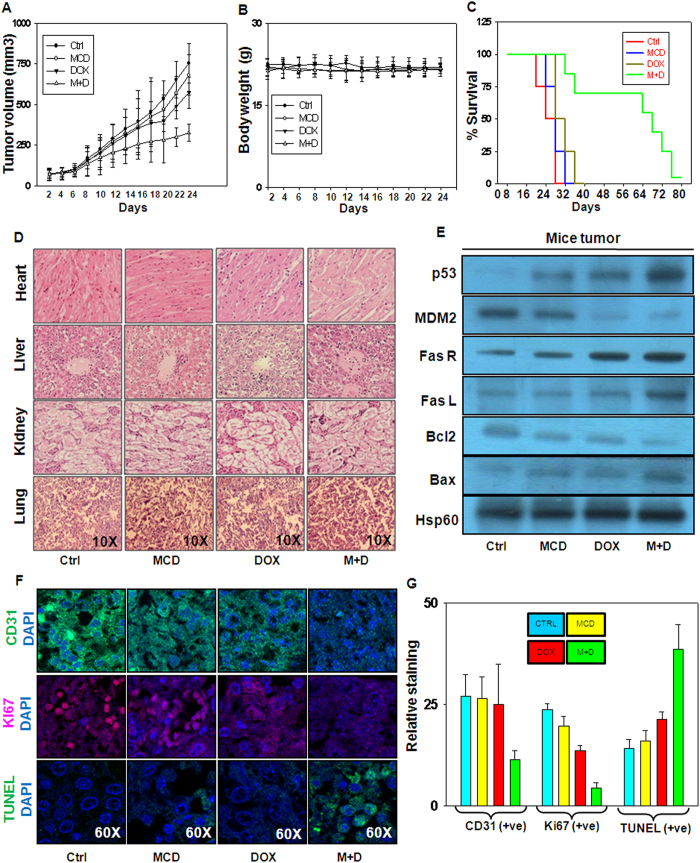
MCD and DOX combination inhibits growth of Hepa1–6 isografted tumors in C57BL/6J mice. Hepa1–6 cell-derived tumors were developed in C57BL/6J mice. Tumor bearing mice were administered normal saline, 64 mg/kg of MCD (i. p./alternative day), 1 mg/kg of DOX (i. p. every alternative day) and a combination of MCD and DOX. (**A**) Tumor initiation and progression (**B**) Changes in body weight (**C**) Median overall survival of mice (**D**) Representative panels of H&E staining of major vital organs such as heart, liver, kidney and lungs of mice (Magnification 10x). (**E**) Western blot analysis of indicated proteins (p53, MDM2, FasR, FasL, Bcl-2, Bax and Hsp60) from tumor lysates of mice administered MCD, DOX and combination of both. (**F**) Representative immunohistochemical analysis of CD31, Ki67 and apoptosis in tumor sections was examined by TUNEL staining. Cell nuclei stained blue with 4’,6-diamidino-2-phenylindole (DAPI) (Magnification 60x). TUNEL assay shows induced apoptotic nucleus (green) in tumors of MCD and DOX together administered mice. (**G**) Bar graph (Mean ± SD) showing the quantitation of average number of CD31, Ki67 and TUNEL positive cells selected from different fields by Image J software. Cropped blots are used in the main figure and full length blots are included in Supplementary figure 6.

We investigated whether p53 is directly involved in the augmentation of DOX induced cell death by MCD. Treatment of MCF-7 and Hepa1–6 cells with inhibitor of p53, pifithrin-α (PFT-α) at 20 μM for 24 h did not exhibit significant cytotoxicity as evaluated by MTT assay (Fig. 4A,D). When cells were pretreated with MCD followed by PFT-α and subsequent treatment of DOX, cell viability significantly increased whereas MCD together with DOX promoted cell death as assessed by MTT assay (Fig. 4B,E). By, long term clonogenic assay, more number of surviving colonies were observed in cells treated with MCD, PFT-α and DOX as compared to MCD plus DOX (Fig. 4C,F). In addition, PFT-α did not play any role in the cellular uptake of DOX as shown by graphical representation of FACS data and bar graph represents mean red fluorescence (Fig. 4G,H). Furthermore, p53 protein levels was significantly diminished and MDM2 protein was increased following co-treatment of MCD and DOX in the presence of PFT-α compared to MCD and DOX treatment. (Fig. 4I and J; p53 and MDM2 panel, highlighted by rectangular block). As FasR/FasL are downstream molecules of p53 their levels was also significantly reduced in MCD, DOX and PFT-α treated cells (Fig. 4I,J; FasR and FasL panel, highlighted by rectangular block). To ascertain the specificity of p53, in regulation of FASR/FasL, MCF-7 cells were transfected with specific p53 siRNA. As shown in Fig. 5K, in the cells transfected with p53 siRNA, the p53 and Fas/FasL protein levels were significantly reduced whereas MDM2 levels were increased in comparison to the control siRNA transfected cells (Fig. 5K; p53 and MDM2 panels, highlighted by rectangular block).

### MCD potentiates the inhibitory effect of DOX on HCC tumorigenesis in mice

To evaluate the combination effect of MCD and DOX on tumor growth, C57BL/6J mice were isografted with Hepa1–6 cells into right flank. When the tumors in all the mice reached to an average volume of approximately 70 mm^3^, the mice were randomly divided into four groups. Tumor bearing mice were administered MCD (64 mg/kg, every alternative days, intraperitoneally), DOX (1 mg/kg, every alternative day 4 h prior to injection of MCD, intraperitoneally), or a combination of both for 18 days (total 9 treatments). Administration of MCD and DOX alone did not inhibit tumor growth, and the tumor growth pattern was similar. However, tumor progressed slowly in mice administered MCD together with DOX and tumor volume was reduced by approximately 50% as compared to tumors in mice administered either agent alone (Fig. 5A). No visible toxicity signs were observed in mice administered MCD, DOX or combination of both as determined by body weight monitoring during the course of the experiment (Fig. 5B). Interestingly, in the mice administered MCD together with DOX, not only tumor volume was reduced but also survivability of mice in this group was also enhanced in comparison with mice in other groups (Fig. 5C). Furthermore, by hematoxylin and eosin (H&E) staining no evident histopathological abnormalities were observed in the vital organs such as heart, liver, kidney and lungs. (Fig. 5D). We also performed western blot analysis in the tumors lysates. It was observed that the levels of p53, FasR, FasL, and Bax were increased whereas decrease in MDM2, and Bcl-2 levels was detected, the pattern being identical to the results obtained by *in vitro* experiments, as shown in Fig. 5E. Furthermore, decrease in the expression of CD31 (endothelial marker) and Ki67 (proliferation marker) was detected by immunohistochemistry (IHC) in the tumor sections of mice administered MCD and DOX together as compared to either agent alone (Fig. 5F; Ki67 and CD31 panel). Finally, to gain insights into whether this combination treatment effectively promotes apoptosis in tumors, sections of tumors were subjected to terminal deoxynucleotidyl transferase dUTP nick end labeling (TUNEL) assay. The increased number of TUNEL positive cells was clearly observed in mice administered by MCD together with DOX as compared to either agent alone (Fig. 5F, TUNEL panel). The quantitative analysis of immunohistochemical staining of CD31, Ki67 and those of TUNEL positive cells are shown in Fig. 5G.

## Discussion

In spite of extensive progress in the treatment of breast and liver cancers, curability rate of these cancers is far from the desired level. Among a variety of chemotherapeutic drugs, DOX is a generalized agent used for treating breast and liver cancer patients. The toxic effect of DOX is a reason for concern that limits its usability. Therefore, the modalities for achieving desired efficacy and preventing high dose toxicity would be beneficial for this therapeutic regimen.

Cell membrane is the key entry point for the drugs and it contains nanometer size microdomain enriched in cholesterol, phospholipids and sphingolipids. Cholesterol, an essential partner of lipid rafts, provides structural stability to cell membrane. Increased cholesterol metabolism and its accumulation have been reported in various cells of breast, prostate and oral cancers^36^,^37^,^38^. Among a variety of exiting cholesterol depleting agents, methyl-β-cyclodextrin (MCD), the most efficient compound used for depleting cholesterol from plasma membrane of cells^39^. MCD has attracted considerable interest in the research area due to its sensitizing effect on various chemotherapeutic drugs. Depletion of membrane cholesterol distorts the integrity of lipid rafts and increases the uptake of ions and small non-electrolyte.

In the present study, we used MCD as a tool to enhance the therapeutic efficacy of DOX, and our data suggest that treatment with DOX and MCD together efficiently potentiates death of MCF-7 and Hepa1–6 cells. MCD depletes cholesterol from the cells (Supplementary Figure 4A-B) and thereby facilitates DOX induced cell death as a consequence of enhancement in intracellular DOX levels. Therefore, MCD augments DOX induced cell death by increasing physiological bioavailability of DOX into the nucleus of cells, and enhances its therapeutic index.

DOX is a potent DNA damaging drug and induces apoptosis in cancer cells via two distinct pathways: membrane associated death receptor pathway by activating caspase-8 and the mitochondrial pathway by activating caspase-9^40^. The membrane associated pathway is the key molecular mechanism of DOX induced cell death which promotes interaction of FasL with FasR, thereby formation of death inducing signalling complex (DISC), leading to cascade activation of different caspases like caspase-8, caspase-3 and caspase-7^41^. Apart from classical apoptotic pathways induced by DOX in cancer cells, it can also induce apoptosis by a new type of mitotic cell death termed as chromosome fragmentation that takes place during the metaphase of cell cycle and occurs in stressed cells. This is distinct from typical apoptosis, independent of caspases and does not demonstrate any typical internucleosomal cleavage of DNA which is not inhibited by overexpression of Bcl-2^42^. In our study, when MCF-7 and Hepa1–6 cells were treated with combination of MCD and DOX, apoptotic death was detected and cells appeared to be under stress. The possibility of mitotic cell death can not be ruled in addition to classical apoptosis in these cells. We found that treatment of cells with MCD and DOX together enhances the secretion of FasL into the culture medium and membrane expression of FasR, indicative of interaction between the two. Significant increase in the cleavage of caspase-8, leading to the increase in caspase-7 activity and ultimately PARP degradation suggests that MCD augments DOX induced apoptosis in MCF-7 and Hepa1–6 cells through extrinsic pathway.

Study by Haupt, S. *et al.*, has shown that tumor suppressor protein p53 activates the extrinsic apoptotic pathway through the induction of genes encoding different membranes proteins such as Fas, DR5 and PERP^43^. p53 works as a nuclear transcription factor and regulates the expression cell cycle proteins, blocks the progression of cell division or induces apoptosis in response to severe DNA damage^44^. It has been reported that wild type p53 sensitizes colorectal carcinoma cells to 5-fluorouracil and topotecan^45^. In the present study, treatment of cells with DOX and MCD together induces severe stress causing enhancement in p53 protein level together with increase in its nuclear localization thereby promoting apoptosis in MCF-7 and Hepa1–6 cells. These results suggest that p53 is a crucial factor for cell death induced by DOX and MCD together. Several other proteins such as FasR and Bax, regulated by p53 in response to chemotherapeutic drugs, are also involved in cell death. Numerous studies have suggested that p53 activation up-regulates FasR as its promoter contains a p53 responsive element, and diminished expression of p53 has been linked with reduction in FasR mediated cell death^46^,^47^,^48^,^49^. Study by Lorenzo, E. *et al.* has suggested that treatment of human primary endothelial cells by DOX enhances the expression of p53 which transcriptionally regulates the expression of FasR^50^. Our *in vitro* results suggested that DOX together with MCD augmented the nuclear accumulation of p53 and increased cell surface expression of FasR in MCF-7 and Hepa1–6 cells. Conversely, silencing of p53 by PFT-α or by siRNA reduced FasR expression, and prevented cell death suggesting that FasR is primary target of p53. Our *in vivo* data demonstrates that treatment of DOX together with MCD significantly retards the tumor growth as compared to either agent alone. Quantitation of DOX in tumor and liver tissue failed to detect DOX except for a presence of very weak intensity of ions signature in kidney tissue of mice administered by MCD together with DOX. It is likely that either the level of DOX is below the detection threshold (~1 ng/ml) or DOX is cleared rapidly from tumor and vital organ (Supplementary Figure 7). Mice administered by MCD together with DOX did not exhibit any toxicity in the major vital organs and the survival of these mice was more as compared to other groups. The reduction in the tumor progression is mainly because of increased expression of membrane protein FasR regulated by p53 in these mice.

In conclusion, the present finding demonstrates that MCD efficiently enhances the toxic effects of DOX through depletion of membrane cholesterol in MCF-7 and Hepa1–6 cells. The proapoptotic function of DOX together with MCD is because of activation of extrinsic apoptotic pathway by p53 resulting in the cleavage of caspases, finally leading to apoptosis. Taken together, these results suggest that combination of low dose of DOX and suboptimal dose of MCD can serve as a potential strategy to minimize side effects and enhances the therapeutic efficacy of DOX in breast and HCC cells.

## Methods

### Drugs, chemicals and antibodies

Methyl β-cyclodextrin (MCD), doxorubicin (DOX), p53 inhibitor pifithrin-α (PFT-α) and methylthioazole-tetrazolium (MTT) and digital vernier caliper were purchased from Sigma-Aldrich (Sigma Aldrich, MO, USA). MCD and DOX were dissolved in water to prepare 100 mM and 1 mM stock respectively and further diluted in culture medium immediately before use. Stock of PFT-α was prepared in DMSO. Antibodies against p53, MDM2, FasR, FasL, Bax, Bcl-2, PARP, Caspase-8, Caspase-7 and Hsp60, mounting medium containing DAPI were purchased from Santa Cruz Biotechnology (Santa Cruz, CA, USA). Collagen-coated chamber slides were purchased from MP Biomedicals, CA, USA.

### Cell culture conditions

MCF-7 (human breast cancer), Hepa1–6 (murine liver cancer) and AML12 (normal hepatocytes) cells were purchased from American Type Culture Collection (ATCC) Manassas, VA, USA and maintained at in-house Cell Repository of National Centre for Cell Science (NCCS), Pune, India. Cells were cultured in Dulbecco’s modified Eagle’s medium (DMEM) with 10% heat inactivated FBS (Hyclone, UT, USA), Penicillin (100 U/ml), Streptomycin (100 μg/ml) (Invitrogen Life Technologies, CA, USA) and incubated at 37 °C in 5% CO_2_ incubator (Thermo Scientific, NC, USA).

### Treatment strategy

Cells were pretreated with MCD for 4 h. Subsequently cells were washed with fresh medium followed by treatment of DOX containing medium for 24 h. In all experiments involving PFT-α, cells were pretreated with MCD for 4 h. Thereafter cells were washed and fresh medium containing PFT-α (20 μM) was added for 1 h prior to the addition of DOX and continuously exposed to the inhibitor during 24 h of treatment.

### MTT (methylthioazole tetrazolium)

Cells (5000/well) were plated in 96-well plate and allowed to grow for 24 h at 37 °C. Next day, cells were treated with varying concentration of DOX for 24 h with or without MCD or PFT-α as illustrated in the treatment strategy. Viability of cells was measured by MTT assay as described^51^.

### Long term clonogenic assay

Cells (5000/well) were plated in 12-well plates and allowed to grow for 24 h and treated as per experimental plan. After 24 h of treatment drug containing medium was changed by fresh medium and cells were allowed to grow for 8–10 days. After completion of experiment, surviving cells were washed with PBS and fixed by 3% paraformaldehyde. The surviving cells were stained with 0.05% crystal violet and images of cells were captured by camera (Olympus, Tokyo, Japan).

### Sandwich ELISA for detection of secreted FasL

Cells (0.3 × 10^6^) were plated in 35 mm culture dishes and treated as described earlier. After 24 h of treatment, medium was collected and secreted FasL was detected in media by sandwich ELISA as described^52^.

### Cholesterol estimation

Cells (0.3 × 10^6^) were plated in 35 mm culture dishes and treated with MCD and DOX as described earlier. Cells were lysed in PBS containing 2% Triton X-100 for 10 min. After centrifugation (12,000 rpm, 15 min), resulting supernatant was used for cholesterol estimation as described^27^.

### FACS analysis of FasR surface staining

Cells (0.3 × 10^6^) were plated in 35 mm culture dishes and allowed to adhere for 24 h. Next day, cells were treated as described earlier followed by harvesting of cells by trypsinization and further processed for FACS analysis for FasR surface staining using FACS Calibur (BD Biosciences, CA, USA ). Briefly, after trypsinization cells were washed three times with PBS. These cells were incubated with primary antibody against FasR for 1h, washed three times with PBS and then incubated with FITC conjugated secondary antibody for 30 min in dark. Flow cytometry analyses were performed using FACS Calibur flow cytometer and the data were analyzed using CellQuest Pro software (BD Biosciences, CA, USA).

### Fluorescence-activated cell sorting (FACS) analysis

Cells (0.3 × 10^6^) were plated in 35 mm culture dishes and allowed to adhere for 24 h. Next day, cells were treated as described earlier followed by harvesting of cells by trypsinization and further processed for FACS analysis using FACS Calibur (BD Biosciences, CA, USA). Briefly, cells were treated with DOX for 24 h with or without MCD or PFT-α as described in the treatment strategy. After 24 h, cells were harvested and washed twice with cold PBS (pH = 7.4) and incubated with Annexin V-FITC in the binding buffer (10 mM HEPES/NaOH, pH 7.5, containing 140 mM NaCl and 2.5 mM CaCl2) for 10 min in the dark at 37 °C. Cells were sorted by FACS Calibur for 10000 events to determine the live and dead cell populations. Data was analyzed using CellQuest Pro software (BD Biosciences, CA, USA).

### Intracellular influx of DOX

Cells (5000/well) were plated in collagen-coated chamber slides, and allowed to adhere for 24 h at 37 °C. Next day, cells were treated with MCD and DOX as described earlier. After completion of experiment cells were washed with PBS and fixed with chilled methanol for 5 min. Cells were stained with DAPI containing mounting medium. Laser scanning confocal microscope (LSM510, Carl Zeiss, Germany) was used to visualize the intracellular accumulation of DOX and photographs were captured. The DOX fluorescence was excited with an argon laser at 488 nm, and the emission was collected through a 550 nm long filter. The uptake of DOX in cells was evaluated by flow cytometry. In brief, cells were cultured in 35 mm culture dishes and were treated with MCD, DOX or PFT-α for 24 h. After 24 h cells were harvested by trypsinization and cells suspension was centrifuged at 2000 rpm for 5 min and resuspended in PBS. Minimum of 50,000 events from each sample was analyzed in order to generate histograms for the fluorescence intensity. Flow cytometry analyses were performed using FACS Calibur flow cytometer and the data were analyzed using CellQuest Pro software (BD Biosciences, CA, USA).

### Immunostaining

Cells (5000/well) were plated in collagen-coated chamber slides, and allowed to adhere for 24 h at 37 ^o^C. Next day, cells were treated with MCD and DOX as described above. After completion of experiment, the cells were washed with PBS and fixed with chilled methanol for 5 min and then blocked by 3% BSA and processed for immunofluorescence analysis as described earlier^53^. Primary antibodies against p53 (1:50) were added and incubated for overnight at humid chamber. Next, cells were washed 3 times with PBS. Fluorescein isothiocyanate (FITC) conjugated secondary antibodies (1:100) were added and incubated for 1 h in a humid chamber. After washing with PBS, mounting medium containing DAPI was added and cells were analysed by confocal microscope and photographed at 60x magnification.

### Whole cell lysate preparation and western blotting

For western blotting following indicated treatments as described earlier, cells were washed thrice with chilled PBS and whole cell lysates were prepared and immunoblotting was performed as described previously^51^.

### p53 siRNA transfection

MCF-7 cells (0.3 × 10^6^) were plated in 35 mm culture dishes and transfected with human specific p53 siRNA as described by Chhipa *et al.*^53^. Cells were further grown for 24 h followed by treatment of MCD and DOX as described previously. Whole cell lysates were prepared for immunoblotting as described previously^51^.

### Mass spectrometric analysis

DOX was extracted from tumor, liver and kidney of mice injected with MCD together with DOX according to described method^54^. DOX was quantified in biological samples by using Agilent 6340 Iontrap (ESI LC-MS, Agilent Technologies, Germany). All data were acquired in positive ionization mode with mass spectrometer operated in Multiple Reaction Monitoring (MRM) in a high resolution mode as described^27^.

### Animal experiments

All animal experiments were performed according to the institutional guidelines, following a protocol approved by the Institutional Animal Ethic Committee (IAEC) of NCCS, Pune, India. Hepa 1–6 cells (5 × 10^6^) were resuspended in 100 μl sterile PBS and subcutaneously injected into right flank of four to five weeks old male C57BL/6J (weight 20 ± 2 g) acquired from experimental animal facility (EAF) of NCCS. When tumors became palpable, tumor bearing mice were arbitrarily divided into four groups each containing six animals (n = 6). Group (a), mice administered with vehicle control, group (b), mice administered MCD (64 mg/kg, intraperitoneally), group (c), mice administered DOX (1 mg/kg, intraperitoneally) and group (d), mice co-administered MCD and DOX. MCD and DOX were dissolved in sterile water and were further diluted with PBS, and then administered into mice. Tumor-sizes were measured every alternative day using Digital Vernier Caliper. Tumor volume (mm^3^) was calculated according to the formula A × B^2^ × 0.52 (A = length; B = width; all parameters in millimeters). After completion of the experiment, the mice were sacrificed by cervical dislocation, and tumors excised for lysates preparation and immunoblotting. For immunohistochemical (IHC) and histopathological studies, sections of tumors and organs were fixed in 10% paraformaldehyde immediately after excision.

### Immunohistochemical and histopathological studies

Fine sections (4–5 μm) were prepared from formalin fixed and paraffin embedded tumor and organs, and fixed on glass slides coated with poly-L-lysine (Safeline Histopathology, Centre, Pune, India). Immunohistochemistry (IHC) and histopathological studies were performed as described previously^26^.

### TUNEL assay

TdT mediated dUTP Nick End Labeling (TUNEL) assay for tumor sections were performed by using APO-DIRECT (BD Biosciences, CA, USA) following the manufacturer’s protocol. Briefly, tumor sections were treated with proteinase K (10 mg/ml) for 30 min. Next, tumor sections were washed three times with PBS and stained with the TUNEL reaction mixture overnight in cold box and washed three times with PBS. The slides were mounted with DAPI containing mounting medium. The slides were visualized by confocal microscopy and TUNEL positive cell were quantify by Image J software.

### Statistical analysis

Statistical comparison was performed by Student’s 2-tailed unpaired t-test by using Sigma Plot software (Systat Software Inc., CA, USA). The values of P < 0.05 were considered statistically significant. Quantitation of colonies was done by using NIH Image J software (Image J Freeware; http://rsb.info.nih.gov/ij/).

## Additional Information

**How to cite this article**: Mohammad, N. *et al.* Strategy to enhance efficacy of doxorubicin in solid tumor cells by methyl-β-cyclodextrin: Involvement of p53 and Fas receptor ligand complex. *Sci. Rep.* **5**, 11853; doi: 10.1038/srep11853 (2015).

## Supplementary Material

Supplementary Information
