# Supplementary material for: Strategy to enhance efficacy of doxorubicin in solid tumor cells by methyl-β-cyclodextrin: Involvement of p53 and Fas receptor ligand complex
Source: Sci Rep. 2015 Jul 7;5:11853. doi: 10.1038/srep11853 (PMC4493576; doi:10.1038/srep11853)
Supplement: Supplementary Information [file srep11853-s1.pdf]

Supplementary figures

Title: Strategy to enhance efficacy of doxorubicin in solid tumor cells by methyl-β-cyclodextrin: Involvement of p53 and Fas receptor ligand complex

Authors: Naoshad Mohammad[1], Shivendra Vikram Singh[1], Parmanand Malvi[1], Balkrishna Chaube[1], Dipti Athavale[1], Muralidharan Vanuopadath[2], Sudarslal Sadasivan Nair[2], Bipin Nair[2] and Manoj Kumar Bhat[1,*]

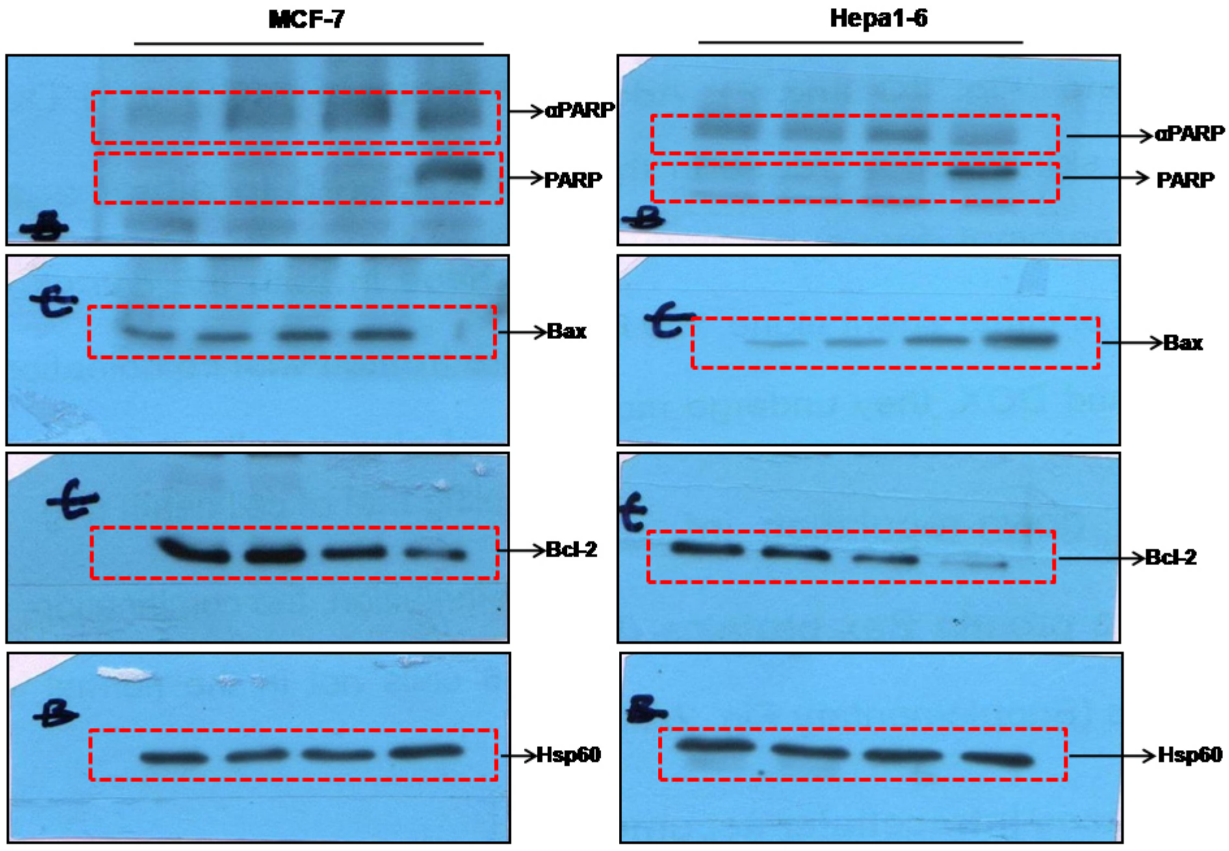

Supplementary figure 1I

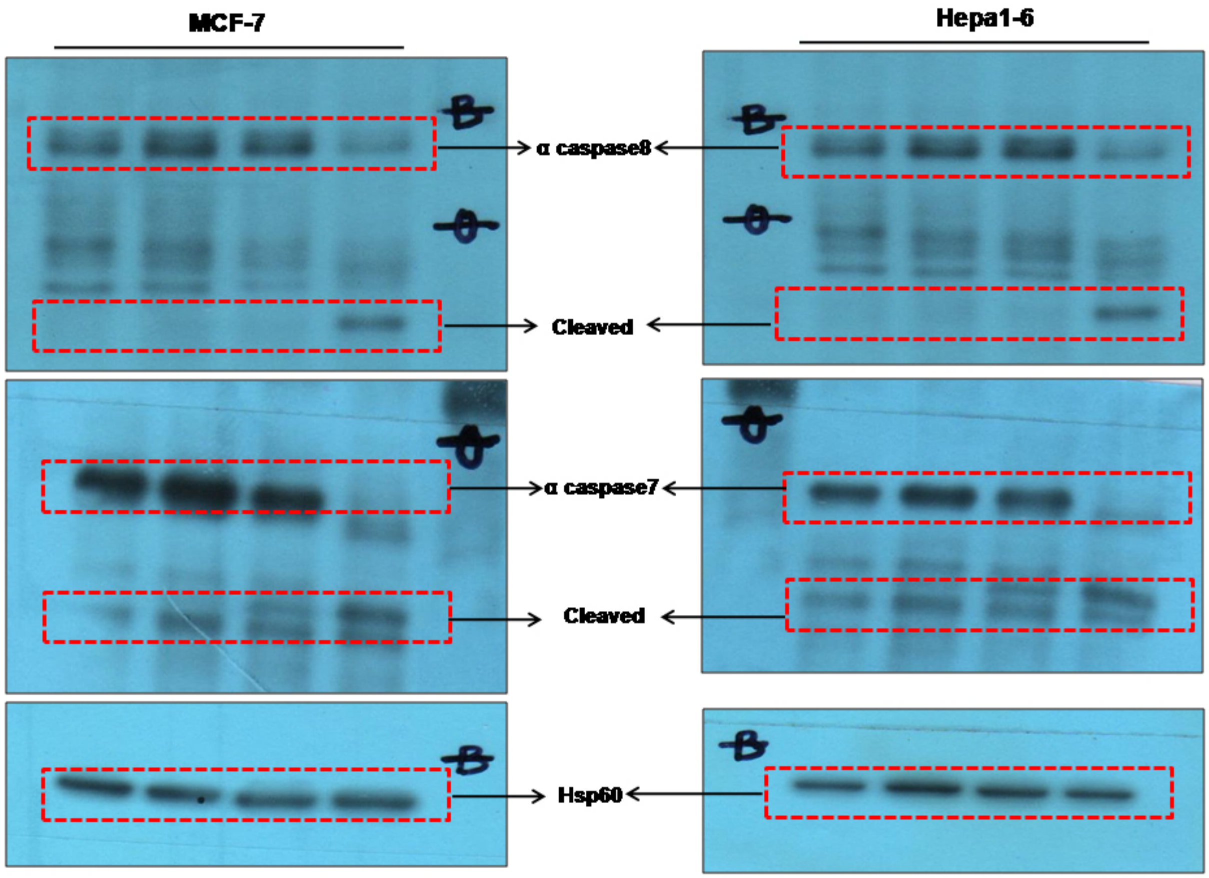

Supplementary figure 1J

Supplementary figure 1. Full length blots of (I) PARP, Bax, Bcl-2 and Hsp60 (J) Caspase 8, Caspase 7 and Hsp60.

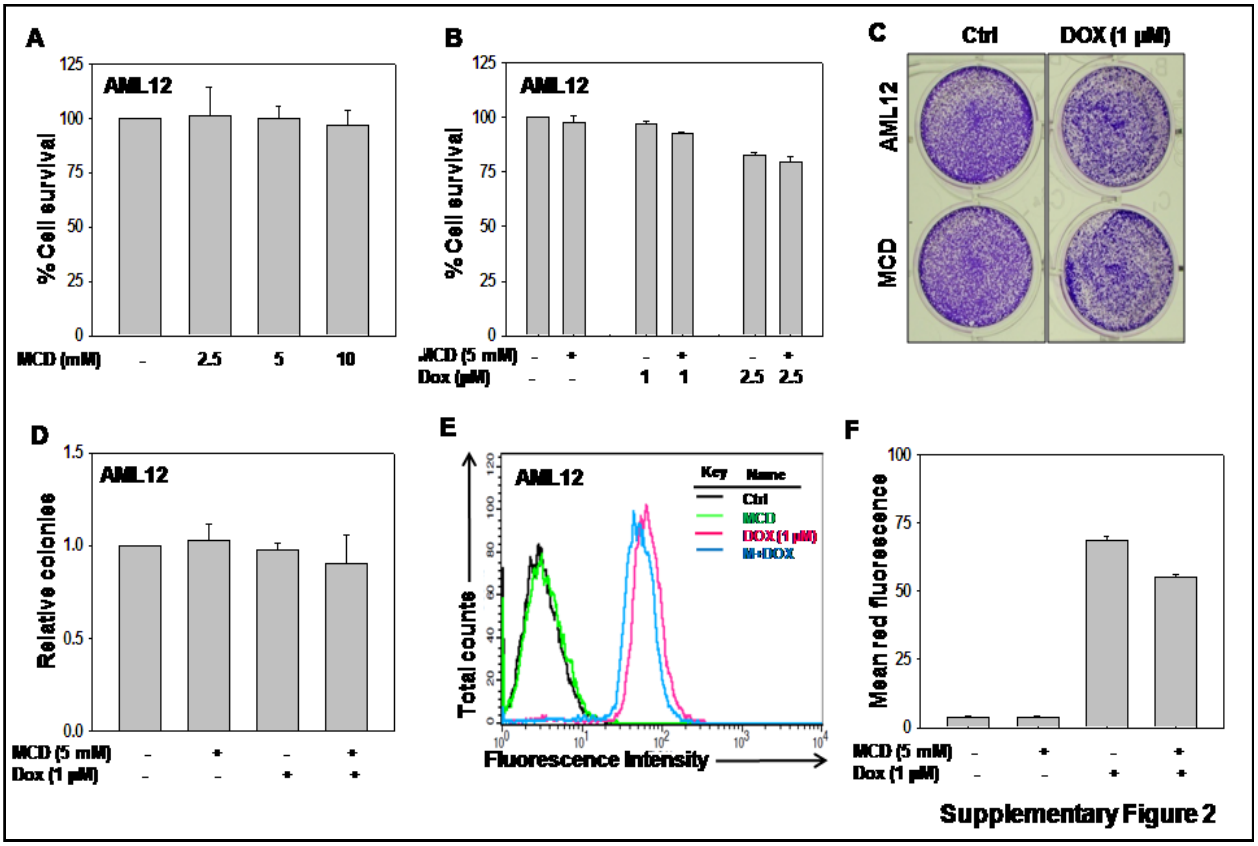

Supplementary figure 2. Treatment with MCD and doxorubicin does not affect survival of AML12 cells (A) Cells were treated with indicated concentration of MCD for 4 h, (B) Cells were treated with indicated concentration of DOX together with MCD for 24 h and cells were subjected to MTT assay. (C) Clonogenic survival assay. (D) Quantitation of colonies by image J software. (E) Representative flow cytometric histogram of intracellular uptake of DOX in AML12 cells. (F) Bar graph is representative of relative quantitation of DOX uptake in AML12 cells. Bar graph represents the Mean ± SD of an experiment done in triplicate.

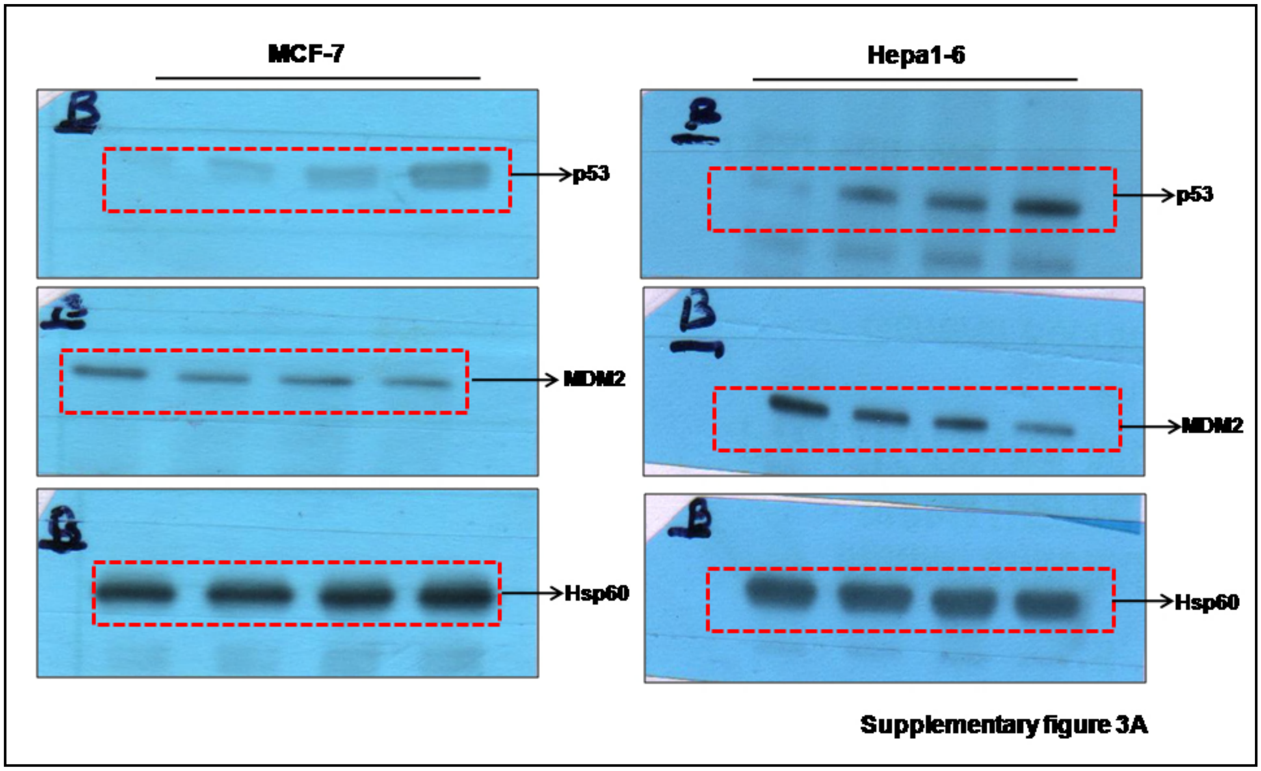

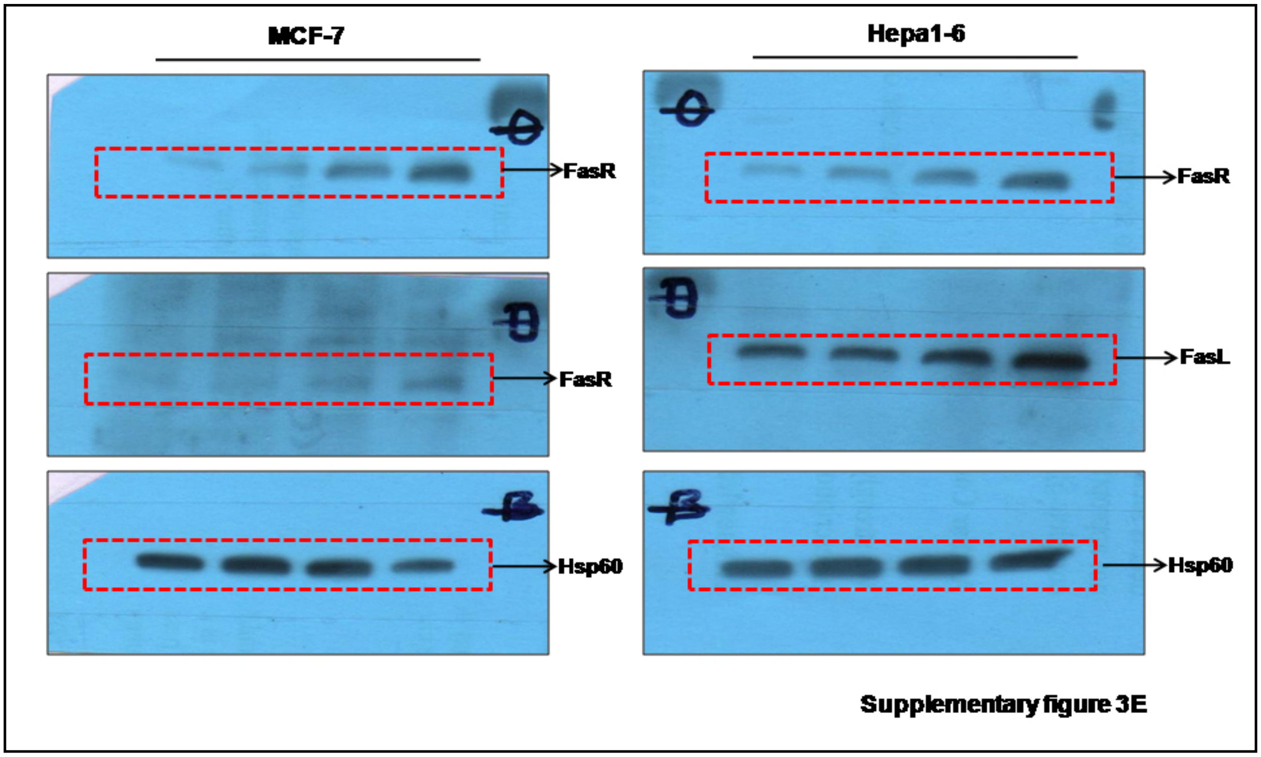

Supplementary figure 3. Full length blots of (A) p53, MDM2 and Hsp60 (E) FasR, FasL and Hsp60.

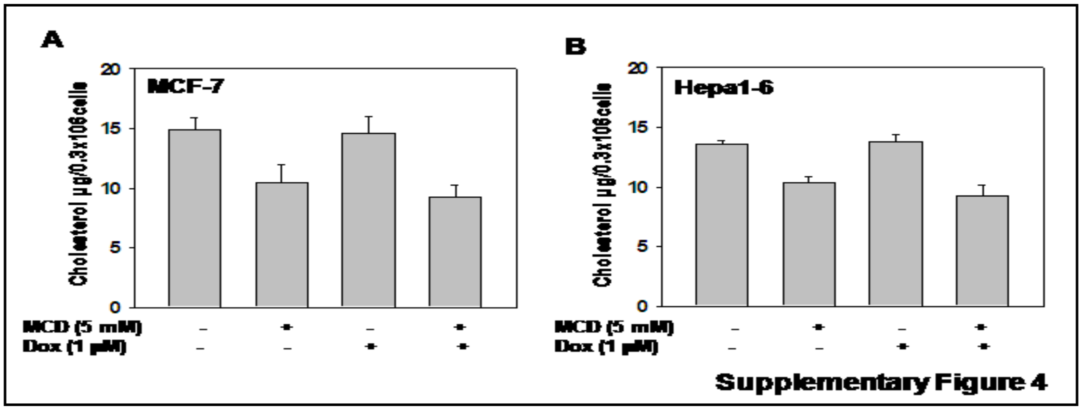

Supplementary figure 4. Total cholesterol (CH) estimation in treated cells. MCF-7 and Hepa1-6 cells were treated with indicated concentration of MCD and DOX and cholesterol was estimated in whole cell extract (A) MCF-7 and (B) Hepa1-6. Bar graph represents the mean ± SD of an experiment done in triplicate.

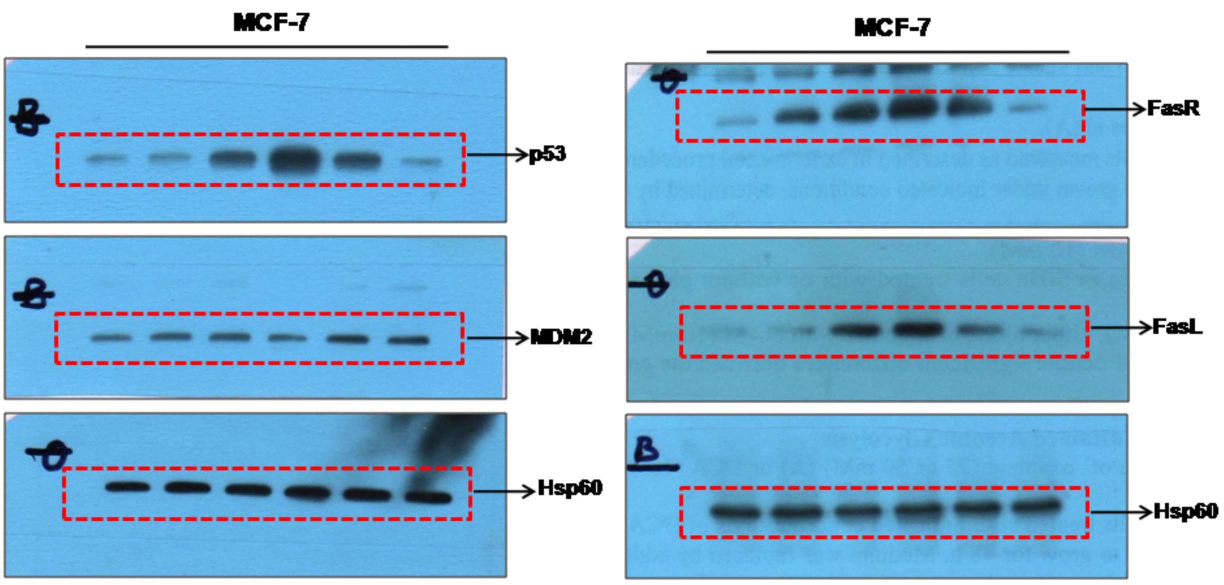

Supplementary figure 5I

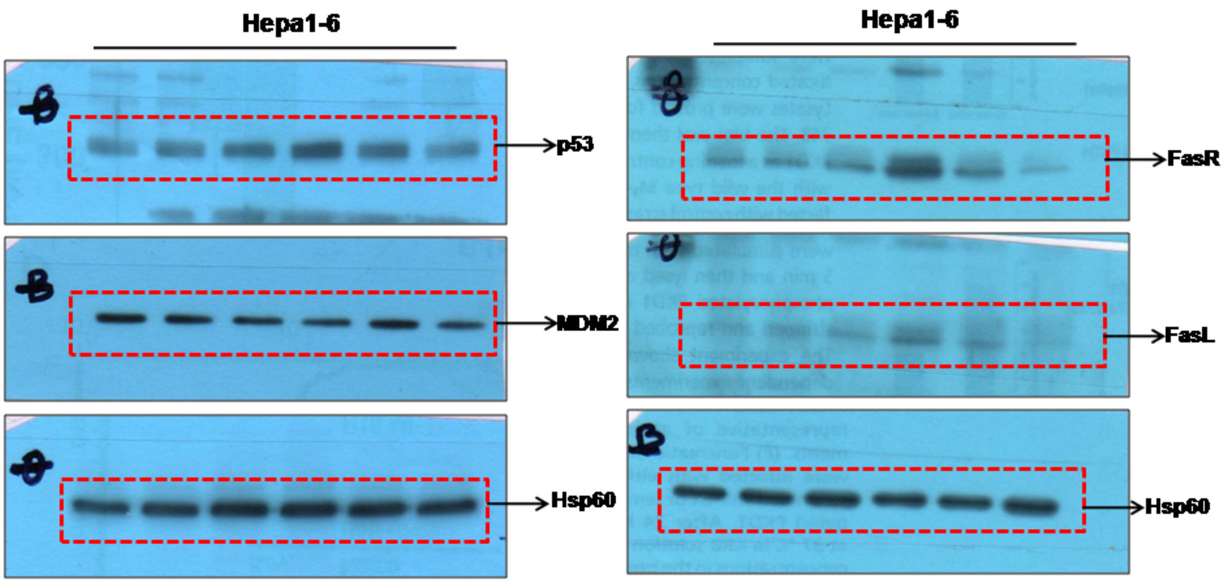

Supplementary figure 5J

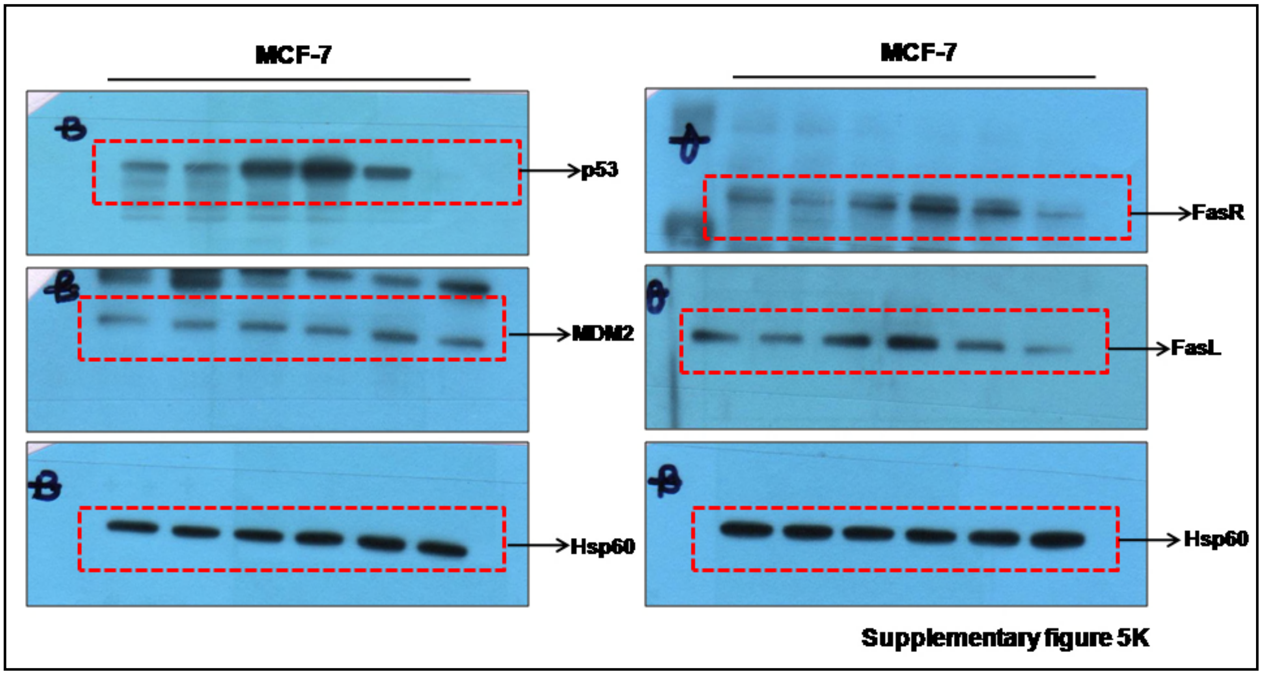

Supplementary figure 5. Full length blots of p53, MDM2, FasR, FasL and Hsp60 (I) MCF-7(J) Hepa1-6 (K) MCF-7 cells transfected with p53 SiRNA.

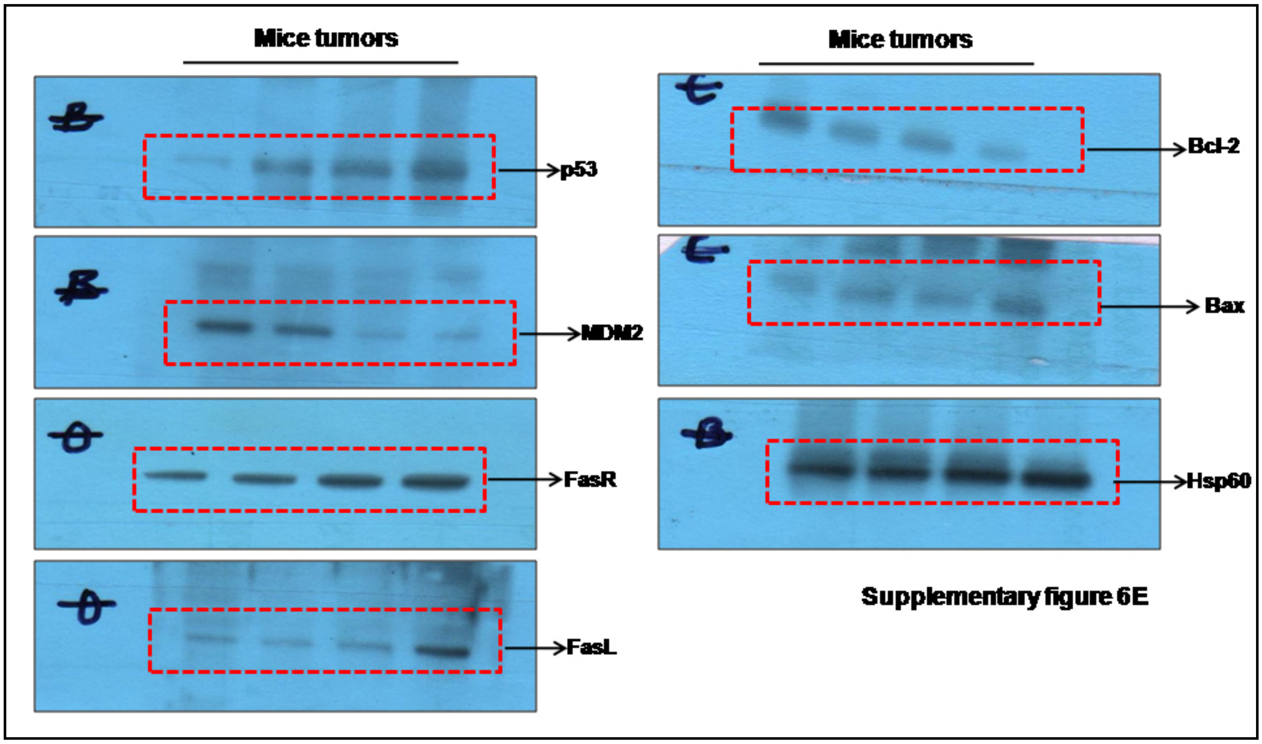

Supplementary figure 6E. Full length blots of p53, MDM2 FasR, FasL, Bcl-2, Bax and Hsp60.

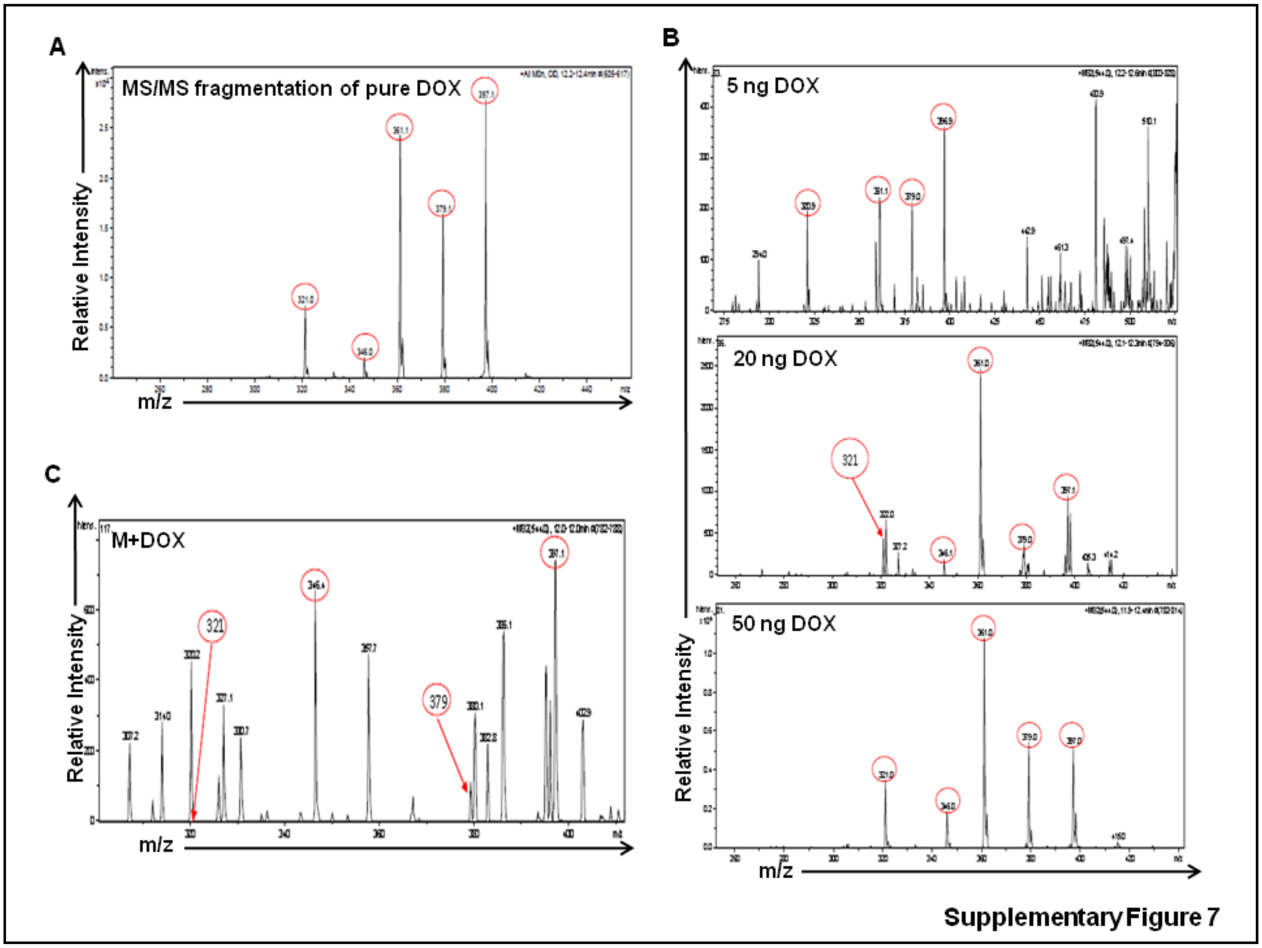

Supplementary figure 7. Detection of DOX in tumor of mice administered with MCD and Dox. (A) MS/MS fragmentation pattern of DOX (B) MS/MS profile of different concentration of DOX (C) MS/MS fragmentation pattern of DOX in the kidney sample of mice administered DOX together with MCD.
